# Seroprevalence of Hepatitis B Virus Carriage Markers Among Students at the University of Yaoundé II, Cameroon

**DOI:** 10.1155/bmri/4866275

**Published:** 2025-10-29

**Authors:** Assiene Oyong Damase Serge, Minyemeck Marielle Flora, Cedric Gueguim, Laurent Akono, Dieudonné Adiogo

**Affiliations:** ^1^ Department of Biology, Higher Institute of Medical Technology of Yaounde, Yaounde, Cameroon; ^2^ Faculty of Medicine and Pharmaceutical Sciences, University of Douala, Douala, Cameroon, univ-douala.com; ^3^ Faculty of Medicine and Biomedical Sciences, University of Yaounde I, Yaounde, Cameroon, uy1.uninet.cm; ^4^ Department of Environmental Sciences, Higher Institute of Agriculture, Wood, Water and the Environment, University of Bertoua, Belabo, Cameroon, univ-bertoua.cm

**Keywords:** hepatitis B, markers, prevalence, students, university

## Abstract

**Background and Objective:**

Cameroon, located in Central Africa, is characterized by a high endemicity of hepatitis B. National studies estimate the prevalence of HBs antigen carriage to range between 8% and 15% in the general population. Identified high‐risk groups include adolescents and young adults, among whom university students represent a vulnerable population. The objective of this study was to assess the seroprevalence of hepatitis B viral biomarkers among students at the University of Yaoundé II in Cameroon.

**Methods:**

We conducted a cross‐sectional analytical study from February 20 to June 20, 2024. Samples were collected at the Soa campus of the University of Yaoundé II. Initial testing was performed using rapid diagnostic tests at the university, followed by ELISA (Fortress Diagnostics) at the blood bank of the Central Hospital of Yaoundé. The detection of anti‐HBs, HBe antigen (HBeAg), anti‐HBe, and anti‐HBc was carried out using the Hightop HBV 5‐in‐1 rapid test. Data were processed and analyzed using Microsoft Excel 2019 and SPSS Version 25. The chi‐square test, Fisher’s exact test, and odds ratio calculations for comparing proportions and logistic regression were used to search for risk factors. The result was significant with a *p* value less than 5%.

**Results:**

A total of 250 students were tested. HBs antigen was positive in eight students (3.2%). Among these, anti‐HBs and HBeAg were absent, while anti‐HBe was positive in six out of eight (75.0%) and anti‐HBc was positive in all eight cases (100%). Only two students (0.8%) had been vaccinated against hepatitis B.

**Conclusion:**

All eight HBsAg‐positive students were in the chronic phase of hepatitis B infection. A history of blood transfusion and unprotected sexual intercourse was strongly associated with HBV infection. Vaccination coverage was extremely low, highlighting the need for targeted immunization programs in this population. Educational and awareness efforts regarding transmission routes and preventive measures must be strengthened.

## 1. Introduction

According to the World Health Organization (WHO), approximately 2 billion people have been exposed to the hepatitis B virus (HBV), with an estimated 1.2 million new infections occurring annually [1]. Chronic hepatitis B, affecting around 254 million individuals worldwide, has become a leading cause of mortality, surpassing HIV, tuberculosis, and malaria [2]. Sub‐Saharan Africa is a region of high endemicity for chronic hepatitis B [3], with prevalence rates ranging from 8% to 20% depending on the country. Cameroon is considered a high‐prevalence zone for hepatitis B surface antigen (HBsAg) carriage and is classified as highly endemic for hepatitis B, with an estimated average prevalence of 10% [4]. Transmission occurs primarily through intrafamilial contact, vertical transmission (mother‐to‐child), and sexual contact—the latter being the predominant route among adolescents and adults [5].

The main serological markers used to assess HBV infection include HBsAg, antibodies to the surface antigen (anti‐HBs), and total antibodies to the core antigen (anti‐HBc). Some studies have also incorporated additional markers such as hepatitis B e‐antigen (HBeAg) and antibodies to the e‐antigen (anti‐HBe) [6].

A study conducted at a university in Brazil reported prevalence rates of 0.136% for HBsAg, 6.44% for anti‐HBc, and 50.8% for anti‐HBs [7]. Among medical students in Uganda in 2005, the overall prevalence was 11.0% for HBsAg and 65.9% for anti‐HBc [8]. Another study involving a cohort of university students in the Central African Republic (CAR) found a high prevalence of HBsAg (15.5%) and anti‐HBc antibodies (42.3%) [9]. In Cameroon, a 2014 study conducted among students at the Faculty of Medicine and Pharmaceutical Sciences of the University of Douala reported an HBsAg prevalence of 5.6% [5].

In this context, we undertook a study to assess the prevalence of HBV markers among students at the University of Yaoundé II, with the aim of contributing to improved management of hepatitis B in this population. The primary objective was to determine the prevalence of viral biomarkers of hepatitis B among students at the University of Yaoundé II–Soa. Specifically, the study is aimed at describing sociodemographic characteristics, determining the prevalence of HBsAg among students, and establishing the profiles of serological markers observed.

The overall goal was to investigate the prevalence of hepatitis B markers among students at the University of Yaoundé II, determine the prevalence of HBV carriage markers, characterize serological profiles, and identify associated risk factors.

## 2. Methodology

### 2.1. Study Population and Design

This was a prospective, cross‐sectional, analytical study conducted at the University of Yaoundé II–Soa during the 2024 academic year. The study spanned a 4‐month period, from February to June 2024.

#### 2.1.1. Selection Criteria

Only students who were officially enrolled at the University of Yaoundé II–Soa and who provided informed consent were included. Students who were enrolled but withdrew during the study period were excluded.

### 2.2. Sampling

A probabilistic sampling technique was employed, involving all students who met the inclusion criteria and agreed to participate during the study period. The sample size (*N*) was calculated using Lorentz’s formula, based on a prevalence rate of 5.4% previously reported among students at the Faculty of Medicine and Pharmaceutical Sciences of the University of Douala. After numerical application, we obtained a minimum sampling of 81 patients. We had 250 patients as the sample size.

#### 2.2.1. Recruitment Strategy and Sample Collection

##### 2.2.1.1. Recruitment Strategy

All eligible students were informed of the study’s objectives and procedures through an official announcement. Those who provided consent were administered a questionnaire and subsequently interviewed. Blood samples were then collected, followed by laboratory analysis.

Data collection was conducted using structured technical forms designed for the study. These forms included sociodemographic information (age, sex, level of education, marital status, etc.), behavioral risk factors, HBsAg screening results, knowledge about hepatitis B, and vaccination status.

Data were gathered through confidential interviews. Each student was approached individually to explain the purpose of the study, verify eligibility criteria, and obtain informed consent.

##### 2.2.1.2. Sample Collection

After the interviews, participants were taken to the laboratory of the Soa Medical and Social Center to ensure privacy and adherence to ethical standards and study requirements.

All students who underwent sample collection met the inclusion criteria. Blood samples were drawn using dry tubes to obtain serum, along with vacutainer needles, sterile gloves, absorbent cotton, and 70% alcohol. Venipuncture was performed at the antecubital fossa.

### 2.3. Sample Analysis

The serum samples obtained from participants were subjected to both rapid diagnostic tests (RDTs) and enzyme‐linked immunosorbent assay (ELISA). HBsAg detection was performed using an RDT followed by ELISA, whereas the other markers were assessed exclusively through RDTs. Only samples that tested positive for HBsAg were further analyzed for additional markers.

#### 2.3.1. HBsAg Screening Using DiaSpot HBsAg One‐Step RDT, United States

##### 2.3.1.1. Principle

This is a qualitative lateral flow immunoassay designed for the detection of HBsAg in whole blood, serum, or plasma. The test strip membrane is precoated with anti‐HBsAg antibodies in the test line region. During testing, the blood/serum/plasma sample reacts with particles coated with anti‐HBsAg antibodies. The mixture migrates upward along the chromatographic membrane via capillary action, where it interacts with the immobilized anti‐HBsAg antibodies, forming a colored line. The appearance of this colored line in the test region indicates a positive result, while its absence indicates a negative result. As a procedural control, a colored line always appears in the control region, confirming that the appropriate sample volume was added and that membrane migration occurred correctly.

#### 2.3.2. Confirmation by ELISA Using the HBsAg Fortress Diagnostics Kit, United Kingdom

##### 2.3.2.1. Principle

The ELISA test is a solid‐phase enzyme immunoassay. Antigens bind to antibodies adsorbed onto the solid support, forming an antigen–antibody complex. A conjugate composed of antihuman immunoglobulin and an enzyme attaches to the target antigen. The immune complex is revealed through an enzymatic reaction in the presence of a colorless chromogenic substrate, which is converted into a colored product. The intensity of the optical density (OD) generated by this reaction is proportional to the concentration of specific antigens.

#### 2.3.3. Detection of Anti‐HBs, HBeAg, Anti‐HBe, and Anti‐HBc Using the Hightop HBV 5‐in‐1 RDT, United States

The principles of the Hightop HBV 5‐in‐1 RDT are as follows:

• *HBsAg/HBeAg*: This reagent is based on the double‐antibody sandwich immunochromatographic principle for detecting HBsAg and HBeAg. If HBsAg or HBeAg is present in the sample, the target antigen binds to colloidal gold‐labeled monoclonal antibodies and is captured by monoclonal antibodies coated on the nitrocellulose membrane (NC). A colored line appears in the test region (T), indicating a positive result. If no colored line appears in the test region, the result is negative.
• *Anti-HBs (HBsAb)*: This reagent uses the double‐antigen sandwich immunochromatographic principle to detect anti‐HBs. If anti‐HBs is present in the sample, the antibody binds to colloidal gold‐labeled recombinant HBsAg and is captured by purified HBsAg coated on the NC membrane. A colored line appears in the test region (T), indicating a positive result. Absence of a colored line indicates a negative result.
• *Anti-HBe/anti-HBc (HBeAb/HBcAb)*: This reagent is based on the competitive immunochromatographic principle for detecting anti‐HBe and anti‐HBc. If the sample contains anti‐HBe or anti‐HBc, no colored line—or only a faint line—appears in the test region (T), indicating a positive result. Conversely, the appearance of two distinct colored lines indicates a negative result.

Regardless of the presence or absence of the target substance, a colored line always appears in the control region (C), serving as a procedural control.

### 2.4. Parameters Studied

The parameters assessed in this study included the following:

• *Sociodemographic data*: age, sex, marital status, region of origin, academic program, and year of study
• *Risk factors*: history of blood transfusion, history of unprotected sexual intercourse, number of partners, use of injectable drugs, tattoos, or piercings
• *Serological status*: HBsAg, anti‐HBs, HBeAg, anti‐HBe, and anti‐HBc
• *Vaccination status against hepatitis B*: number of doses received
• *Serological testing for HBsAg*: performed using the DiaSpot RDT followed by ELISA

### 2.5. Data Processing

The data collected using the technical forms were processed and analyzed under the supervision of a statistician, using Microsoft Excel 2019 and SPSS Version 25.

The statistical tests applied included the chi‐square test, Fisher’s exact test, and odds ratio calculations for comparing proportions. Logistic regression was used to identify associated risk factors. Results were considered statistically significant at a *p* value less than 0.05.

### 2.6. Ethical Considerations

We obtained ethical clearance from the national ethics committee of the University of Douala (No. 4595 CEI‐Udo/07/2024/M) and authorization from the authorities of the University of Yaoundé II in Cameroon (No. 24/07/UYII/CAB/R) and the Central Hospital of Yaoundé (No. 145/24/AP/MINSANTE/SG/DHCY/CM/SM).

The confidentiality of the analyses was ensured during and after the study by all those involved in the study through the coding of participant data.

## 3. Results

**Figure 1:**
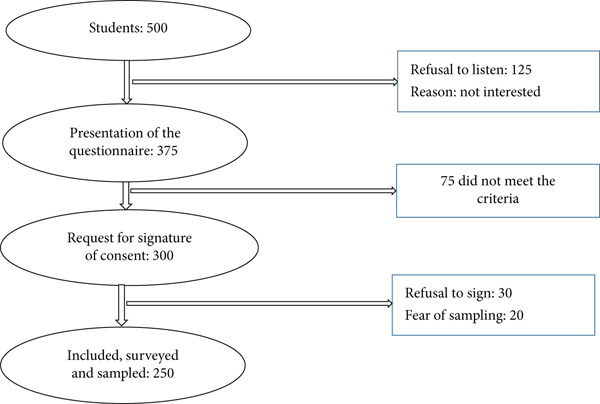
Summary of student recruitment during the study.

During the study period, a total of 500 students were approached. Of these, 250 agreed to participate and met the inclusion criteria, resulting in a participation rate of 50% (Figure 1).

### 3.1. Sociodemographic Characteristics

#### 3.1.1. Age Distribution

**Figure 2:**
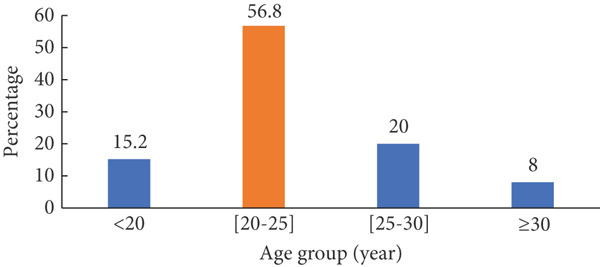
Breakdown of the population by age group.

At the end of the recruitment period, 250 participants were enrolled. Their ages ranged from 18 to 30 years, with a mean age of 23.0 ± 3.4 years. The majority of participants were between 20 and 25 years old (Figure 2).

#### 3.1.2. Sex Distribution

**Figure 3:**
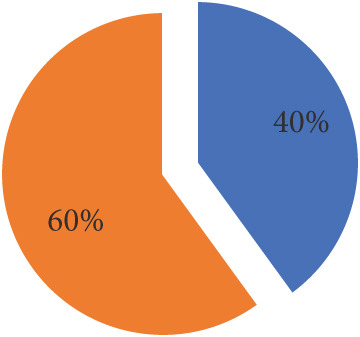
Breakdown of the population by gender.

Among the recruited participants, males accounted for 60% (*n* = 150), resulting in a male‐to‐female sex ratio of 1.5 (Figure 3).

### 3.2. HBsAg Carriage

A total of 250 students were tested. HBsAg was positive in eight students, representing a prevalence of 3.2%. Among these HBsAg‐positive individuals, anti‐HBs and HBeAg were absent, while anti‐HBe was detected in six out of eight (75.0%) and anti‐HBc was positive in all eight cases (100%). Only two students (0.8%) had been vaccinated against hepatitis B.

HBsAg was positive in eight students, corresponding to a prevalence of 3.2% (see Table 1). The results also showed that male sex was significantly associated with HBV carriage, as all HBV‐positive participants were male. This difference was statistically significant (*p* = 0.023) (Table 2). The HBV carriers were distributed across academic levels as follows: second year of undergraduate studies (4.5%), third year of undergraduate studies (5.3%), first year of master’s program (3.2%), and first year of doctoral studies (9.1%) (*p* = 0.391).

**Table 1:** Prevalence of hepatitis B markers.

	**Frequency**	**Percentage (%)**
HBsAg positive (*n* = 250)	8	3.2
Anti‐HBsAb positive (*n* = 8)	0	0.0
Anti‐HBeAg positive (*n* = 8)	0	0.0
Anti‐HBeAb positive (*n* = 8)	6	75.0
Anti‐HBcAb positive (*n* = 8)	8	100

**Table 2:** Frequency of hepatitis B according to age, sex, marital status, number of sexual partners, and region of origin.

**Variables**	**HBsAg**		**OR (CI 95%)**	**p** **value**
	**Positive (%)**	**Negative (%)**		
Average age (year)	25.6 ± 2.7	22.9 ± 3.5	1.24 (1.02–1.51)	0.032
Sex				0.023
Female	0 (0.0)	100 (100)	—	
Male	8 (5.3)	142 (94.7)	—	
Marital status			—	1.0
Single	8 (3.3)	237 (96.7)	—	
Married	0 (0.0)	5 (100)	—	
Number of partners			—	1.0
0	0 (0.0)	53 (100)	—	
1	8 (4.7)	162 (95.3)	—	
2	0 (0.0)	24 (100)	—	
3	0 (0.0)	3 (100)	—	
Region of origin				0.003
Center	3 (2.7)	110 (97.3)	—	
East	0 (0.0)	6 (100)	—	
Far North	2 (40.0)	3 (60.0)	—	
Coastline	0 (0.0)	18 (100)	—	
North	0 (0.0)	6 (100)	—	
North West	0 (0.0)	7 (100)	—	
West	2 (2.7)	72 (97.3)	—	
South	1 (5.0)	19 (95.0)	—	
Southwest	0 (0.0)	1 (100)	—	

### 3.3. Biological Parameters

Among the 250 participants, 8 tested positive for HBsAg, corresponding to a prevalence of 3.2%. Anti‐HBs and HBeAg were absent in all eight HBsAg‐positive individuals, while anti‐HBe was detected in six out of eight (75.0%) and anti‐HBc was positive in all eight cases (100%) (see Table 1).

### 3.4. Risk Factors for HBsAg Carriage

Medical histories revealed that 13.3% (*n* = 2) of HBsAg‐positive students had received a blood transfusion and reported a history of jaundice. Additionally, 4.1% (*n* = 6) of HBsAg‐positive students reported engaging in unprotected sexual intercourse (Table 3).

**Table 3:** Frequency of hepatitis B according to risk factors.

**Variables**	**HBsAg**		**OR (CI 95%)**	**p** **value**
	**Positive (%)**	**Negative (%)**		
Blood transfusion				0.041
Yes	2 (13.3)	13 (86.7)	5.87 (1.08–31.98)	
No	6 (2.6)	229 (97.4)	1	
Drug injection				1.0
Yes	0 (0.0)	1 (100)	—	
No	8 (3.2)	241 (96.8)	—	
Sharing injection equipment				1.0
Yes	0 (0.0)	1 (100)	—	
No	8 (3.2)	241 (96.8)	—	
Tattoo/piercing			—	1.0
Yes	0 (0.0)	20 (100)	—	
No	8 (3.5)	222 (96.5)	—	
Unprotected report				0.344
Yes	6 (4.1)	140 (95.9)	2.19 (0.43–11.05)	
No	2 (1.9)	102 (98.1)	1	
Vaccination				1.0
Yes	0 (0.0)	2 (100)	—	
No	8 (3.2)	240 (96.8)	—	

### 3.5. Hepatitis B Vaccination

A total of 0.8% (*n* = 2) of students had been vaccinated against hepatitis B (received all three doses), while 99.2% remained unvaccinated.

## 4. Discussion

### 4.1. Sociodemographic Characteristics

A total of 250 students were recruited, with ages ranging from 18 to 30 years and a median age of 23. The most represented age group was between 20 and 25 years. Male participants predominated, accounting for 60% (*n* = 150), while females represented 40% (*n* = 100). These findings are consistent with studies conducted by Bouba et al. in the Far North region of Cameroon, which reported a male participation rate of 59.7%, and Barry et al. in Guinea, which found similar proportions among blood donors—60% male and 40% female [10; 11].

In our study, 54.4% (*n* = 136) of participants were enrolled in the Faculty of Law and Political Science. Undergraduate students represented 73.2% (*n* = 183), a result that aligns with findings by Eloumou Bagnaka et al., who reported a similar but slightly higher frequency of 76.6%, likely due to a larger sample size [5].

### 4.2. Biological Parameters

The prevalence of HBsAg among students was 3.2%. This figure is considerably lower than those reported by Eloumou Bagnaka et al. in Cameroon (5.6%) and by Tesfa et al. in Ethiopia (11.5%) among medical students [6; 12]. This discrepancy may be attributed to the fact that medical students represent a higher risk population due to their constant exposure to patients in clinical settings and also to the larger sample sizes used in those studies.

However, the prevalence observed in our study was higher than that reported among students in Brazil by Pinto et al. (0.136%) and in Pakistan by Mawouma et al. (2.13%) [7; 13]. This difference reflects Cameroon’s classification as a high endemicity zone for hepatitis B, in contrast to Brazil (low endemicity) and Pakistan (intermediate endemicity) [10, 11].

Among HBsAg‐positive students, the prevalence of anti‐HBc was 100%. This result differs from the 6.44% prevalence reported at the Urban University of Rio de Janeiro in Brazil by Pinto et al. [12]. The discrepancy is likely due to the fact that, in our study, this marker was assessed only in HBsAg‐positive individuals.

The prevalence of HBeAg among HBsAg‐positive students was zero, indicating that none of the participants were in the viral replication phase of hepatitis B. This finding contrasts with that of Njoya et al., who reported HBeAg positivity among pregnant women and healthcare personnel in Yaoundé. The similarity in hepatitis B management protocols across different groups in Yaoundé may explain this concordance [4]. However, our result differs from that of Mawouma et al., who found a higher prevalence of 13.7% among pregnant women in the Far North region of Cameroon [13].

### 4.3. Correlations Between Hepatitis B Prevalence, Sociodemographic Factors, and Risk Factors

HBV‐positive students were older (mean age: 25.6 years) compared to HBV‐negative students (mean age: 22.9 years). This difference may be explained by the fact that younger individuals have benefited from childhood vaccination programs, which were only introduced in Cameroon in the early 2000s. This finding aligns with that of Mawouma et al., who reported similar results among pregnant women in the Far North region of Cameroon [13].

Male sex was associated with HBV carriage, as all HBV‐positive students were male. This may be due to the fact that women generally have better access to healthcare services, including screening during prenatal care, which facilitates earlier detection and management. This result is consistent with a study conducted in Benin by Aboudou et al., which showed that males were twice as likely to carry HBV compared to females [14].

Among HBV‐positive students, some had a history of blood transfusion. This may be attributed to limitations in blood screening and safety procedures in Cameroon, particularly in rural and remote areas. Similar associations between transfusion history and HBsAg carriage were reported by Noubiap et al. and Ousmane et al. [15, 16].

Other HBV‐positive students reported engaging in unprotected sexual intercourse. This may be explained by the fact that students, due to their young age and socially active lifestyles, are less likely to use protection during sexual activity. A lack of experience and awareness about transmission risks may contribute to these behaviors. A study conducted in Côte d’Ivoire by Toumin et al. identified sexual activity as a significant risk factor for HBV carriage [17]. These findings are also consistent with those of Eloumou Bagnaka et al., who reported blood transfusion and unprotected sex as major risk factors among HBsAg‐positive students [5].

### 4.4. Study Limitations

This study presents certain methodological limitations that warrant consideration. The investigation of hepatitis B serological markers was conducted exclusively among patients who tested positive for the surface antigen (HBsAg). Consequently, individuals who were HBsAg‐negative were not assessed for other markers such as anti‐HBc antibodies or HBV DNA. This approach narrows the scope of the study, particularly in identifying cases of occult hepatitis B or atypical serological profiles. It also limits the broader understanding of the infection’s dynamics by excluding phases of resolution or partial immune response. Moreover, this selection may introduce a bias, reducing the generalizability of the findings to the wider population exposed to the virus. Further studies including all serological profiles are needed to provide a more comprehensive assessment of HBV circulation.

## 5. Conclusion

The prevalence of HBsAg among students at the University of Soa was 3.2%. Among those who tested positive, the prevalence of HBeAg and anti‐HBs was zero, while anti‐HBc and anti‐HBe were present in 100% and 75% of cases, respectively. All HBsAg‐positive students were in the chronic phase of hepatitis B infection.

Identified risk factors included blood transfusion and unprotected sexual intercourse. Additionally, vaccination coverage was extremely low (0.8%).

These findings underscore the urgent need for targeted hepatitis B vaccination programs within this population. Educational and awareness efforts regarding transmission routes and preventive measures must also be strengthened.

**Nomenclature**
HBV: hepatitis B virus
HBsAg: hepatitis B surface antigen
Anti‐HBc: anti–hepatitis B core antibody
Anti‐HBs: anti–hepatitis B surface antibody
Anti‐HBe: anti–hepatitis B extractable antibody
HBeAg: hepatitis B extractable antigen
WHO: World Health Organization

## Ethics Statement

Informed consent was obtained from all individuals. The study was approved by national ethics committee of the University of Douala.

## Disclosure

All the authors of this work agree to this publication.

## Conflicts of Interest

The authors declare no conflicts of interest.

## Author Contributions

A.O.D.S. and M.M.F.: conceptualization, investigation, methodology, formal analysis, writing—original draft, and writing—review and editing. C.G.: methodology and writing—original draft. L.A.: formal analysis and writing—original draft. D.A.: supervision, methodology, and writing—original draft.

## Funding

No funding was received for this manuscript.

## Data Availability

Data will be made available on request.
